# Patient-Derived Transcriptomics Reveals Distinct IFNγ-Associated Myeloid States and Mitochondrial Complex I Programs in Rheumatoid Arthritis and Osteoarthritis

**DOI:** 10.3390/biomedicines14092094

**Published:** 2026-09-17

**Authors:** Heekyong R. Bae, Byeongchan Son, Seong-Su Choi, Hye-Bin Jeong, Howard A. Young, Eun-Young Kwon

**Affiliations:** 1 Center for Food and Nutritional Genomics, Kyungpook National University, Daegu 41566, Republic of Korea; 2 Department of Food Science and Nutrition, Kyungpook National University, Daegu 41566, Republic of Korea; 3 Department of Biology, Kyungpook National University, Daegu 41566, Republic of Korea; 4 Department of Statistics, Kyungpook National University, Daegu 41566, Republic of Korea; 5 Cancer Innovation Laboratory, Center for Cancer Research, National Cancer Institute, Frederick, MD 21702, USA; 6 Center for Beautiful Aging, Kyungpook National University, Daegu 41566, Republic of Korea

**Keywords:** rheumatoid arthritis, osteoarthritis, synovial macrophages, synovial monocytes, interferon-gamma, mitochondrial Complex I, mitochondrial translation, immunometabolism

## Abstract

**Background/Objectives:** Macrophages are key contributors to the pathogenesis of rheumatoid arthritis (RA) and osteoarthritis (OA), but their disease-associated immunometabolic states remain incompletely understood. **Methods:** Here, we comparatively analyzed patient-derived transcriptomic data from synovial tissues and synovial macrophages in RA and OA. **Results:** RA synovial tissues showed stronger interferon-γ (IFNγ)-associated signatures and reduced mitochondrial Complex I-related programs compared with OA. Similar reductions in oxidative phosphorylation, mitochondrial translation, and Complex I-related transcription were observed in synovial fluid-derived RA macrophages relative to control macrophages. In an independent dataset of synovial tissue-derived OA macrophages, inflammatory-like OA macrophages exhibited concurrent enrichment of glycolytic and oxidative metabolic programs. Bulk RNA-seq analysis of synovial monocytes within a common study framework further showed that reduced mitochondrial translation and Complex I-related programs were preferentially associated with leukocyte-rich inflammatory RA. At single-cell resolution, IFN-responsive monocytes enriched in leukocyte-rich RA showed reduced transcriptional representation of both nuclear- and mtDNA-encoded Complex I components. **Conclusions:** Collectively, these findings link an IFN-responsive inflammatory myeloid state in RA with coordinated suppression of mitochondrial translation and Complex I-related transcription, suggesting a transcriptional state that may increase susceptibility to mitochondrial Complex I dysfunction during persistent inflammation.

## 1. Introduction

Rheumatoid arthritis (RA) is a female-predominant systemic autoimmune disease characterized by inflammation of multiple joints, leading to progressive joint destruction [1,2]. In contrast, osteoarthritis (OA) is a degenerative joint disease caused primarily by mechanical stress or injury, with aging-associated changes, such as chondrocyte senescence and immune aging, thought to accelerate its pathogenesis [3]. RA is characterized by systemic inflammation, whereas OA involves localized, low-grade inflammation. The effectiveness of anti-inflammatory treatments in RA underscores the central role of inflammatory responses in its pathogenesis [4]. RA synovial macrophages frequently exhibit M1-associated pro-inflammatory transcriptional features, including expression of TNF-α, IL-1β, and IL-6 [5]. However, recent research has shown that early innate inflammation, such as macrophage activation, is also associated with OA pathogenesis [6]. Therefore, a better mechanistic understanding of disease-associated myeloid responses is needed to distinguish the inflammatory processes underlying RA and OA.

Interferon-gamma (IFNγ) acts as a highly pleiotropic “double-edged sword” in joint pathology, exerting both protective and deleterious effects in rheumatoid arthritis [7]. For instance, IFNγ induces inflammatory and tissue-destructive responses in articular chondrocytes and osteoblasts through protein kinase R (PKR) activation [8] while another report demonstrated that the absence of IFNγ signaling, via the genetic ablation of interferon-gamma receptor 1 (IFNγR1), impaired cartilage regeneration in a full-thickness cartilage injury (FTCI) mouse model [9]. Current evidence suggests that IFNγ exerts context-dependent regulatory effects in RA development [10], including modulation of Th17-associated autoimmune responses [11,12]. However, increased local IFNγ signaling during early RA has also been associated with macrophage-mediated joint inflammation and stress-related immune activation within the synovial microenvironment. On the other hand, in OA, experimental studies suggest that appropriate IFNγ signaling contributes to macrophage-mediated debris clearance and subsequent cartilage repair during early-stage injury responses [9,13]. Thus, a deeper mechanistic understanding of the context-dependent roles of IFNγ may be essential for the development of more effective and disease stage-specific therapeutic strategies.

Macrophage dysfunction is considered a key factor in the development and progression of RA [14,15,16]. M1-like macrophage activation has traditionally been associated with reduced oxidative phosphorylation (OXPHOS) and increased reliance on glycolysis [17]. When M1 macrophages are activated, they produce reactive oxygen species (ROS). In high pro-inflammatory conditions, excessive mitochondrial ROS production damages mitochondrial DNA (mtDNA), and mtDNA release can activate the cyclic GMP-AMP synthase (cGAS) and stimulator of interferon genes (STING) axis, perpetuating type I IFN production [18,19]. This mechanism has been linked to alterations in mitochondrial dynamics, such as mitochondrial fission and mitophagy, leading to mitochondrial dysfunction [20,21]. Therefore, understanding the interplay between mitochondrial dysfunction and macrophage-mediated inflammation is crucial for identifying novel therapeutic strategies to target chronic inflammation in RA.

Mitochondrial complex I (CI), also known as NADH:ubiquinone oxidoreductase, is the first enzyme in the electron transport chain (ETC) and plays a critical role in energy production and redox balance in mitochondria. It directly modulates ROS generation, which may occur under normal function or when the complex is inhibited [22,23]. Under physiological conditions, a small fraction of electrons leaking from CI during respiration can reduce molecular oxygen to form superoxide (O_2_^−^) [22]. This ROS production is minimal and tightly regulated by mitochondrial antioxidant systems such as superoxide dismutase (SOD) [24]. Under stress conditions, reverse electron transport toward Complex I can generate substantial ROS [25], a mechanism recognized as a major source of ROS in M1 macrophages. However, CI itself is highly susceptible to oxidative damage and may become a direct target of excessive ROS accumulation. In particular, oxidative stress can damage the iron–sulfur (Fe–S) clusters required for electron transfer within CI, thereby impairing CI function and mitochondrial respiration [26].

Here, we investigated the mechanistic differences between RA and OA synovial macrophages through comparative immunometabolic analyses. Rather than viewing RA simply as a condition of amplified inflammatory signaling relative to OA, we sought to explore whether RA synovial macrophages exhibit a distinct immunometabolic state associated with altered mitochondrial Complex I-related transcription and persistent IFN-responsive signaling. In particular, our analyses aimed to examine whether IFN-responsive states are associated with mitochondrial translation and Complex I-related transcriptional alterations in RA synovial myeloid cells.

## 2. Materials and Methods

### 2.1. Comparative Transcriptomic Analysis of Synovial Tissue in RA and OA Patients

To investigate gene expression profiles in synovial tissue from RA and OA patients compared to healthy controls, we utilized datasets GSE1919 and GSE55457 from the Gene Expression Omnibus (GEO). Both datasets were analyzed using GEO2R, which applies linear modeling and *t*-tests to assess differential expression between selected groups. For GSE1919, 15 synovial tissue expression profiles were analyzed, including five healthy control, five RA, and five OA samples. Within both the RA and OA groups, the five arrays comprised samples from three individual patients and two patient pools [27]. Expression profiling was conducted using the Affymetrix Human Genome U95A Array Platform. For GSE55457, 33 synovial tissue samples were analyzed, comprising 10 healthy controls, 13 RA samples, and 10 OA samples [28]. Expression profiling was performed using the Affymetrix Human Genome U133A Array Platform. We performed differential expression analysis using GEO2R to examine gene expression differences among RA, OA, and control samples, facilitating the exploration of molecular signatures specific to each condition.

### 2.2. Transcriptomic Profiling of Synovial Fluid-Derived Macrophages in RA Patients

To examine gene expression specifically in synovial fluid-derived macrophages from RA patients, we utilized the GSE97779 dataset from GEO. This dataset includes 14 samples, consisting of 9 synovial fluid-derived RA macrophages freshly isolated from the synovial fluid of RA patients and 5 control macrophages derived from primary human monocytes cultured with a macrophage colony-stimulating factor (M-CSF) for 3 days. Gene expression profiling was performed using the Affymetrix Human Genome U133 Plus 2.0 Array Platform [29]. We conducted differential expression analysis using GEO2R to compare synovial fluid-derived RA macrophages with control macrophages. GEO2R is an online analysis tool that utilizes the limma package in R for linear modeling and statistical testing. Genes were considered significantly differentially expressed based on an adjusted *p*-value < 0.05 after Benjamini–Hochberg correction for multiple testing [30].

### 2.3. Transcriptomic Profiling of Synovial Tissue-Derived Macrophages in OA Patients

To independently examine inflammatory and metabolic transcriptional programs within synovial tissue-derived OA macrophages, we analyzed GSE123492. This dataset contains RNA-sequencing profiles of FACS-purified macrophages isolated from synovial tissue. The original RNA-seq cohort comprised 12 arthritis samples, including nine OA and three inflammatory-arthritis samples, with one OA sample excluded as an outlier in the original study [31]. The iOA-versus-cOA analysis was performed using the OA subgroups defined by Wood et al.

### 2.4. Bulk and Single-Cell RNA-Seq Profiling of Synovial Monocytes in RA and OA Patients

Accelerating Medicines Partnership Rheumatoid Arthritis/systemic Lupus Erythematosus (AMP RA/SLE) Phase I bulk and single-cell RNA-seq data were reanalyzed to compare RA and OA synovial monocytes within a common study framework [32]. RA samples were classified as leukocyte-rich or leukocyte-poor according to the inflammatory phenotype defined in the original AMP study. Bulk RNA-seq analysis included QC-passed sorted synovial monocytes from 16 leukocyte-rich RA, 14 leukocyte-poor RA, and 13 OA samples. Genes were ranked by Welch’s *t*-statistic and analyzed by pre-ranked GSEA using the same predefined mitochondrial gene sets as in the preceding analyses. NES, permutation-derived *p*-values, and Benjamini–Hochberg-adjusted FDR values were calculated. Because the processed matrix lacked mitochondrially encoded genes, bulk pathway analyses reflected nuclear-encoded gene-set members. Single-cell analyses used the published AMP annotations without re-clustering and included 750 monocytes from 9 leukocyte-rich RA, 3 leukocyte-poor RA, and 2 OA donors: SC-M1 *(n* = 349), SC-M2 (*n* = 237), SC-M3 (*n* = 93), and SC-M4 (*n* = 71). Complex I-related transcription was assessed using a 39-gene nuclear NDUF set (Supplementary Table S1) and the seven mtDNA-encoded MT-ND genes. IFN-responsive transcription was assessed using an 11-gene IFNγ-associated transcriptional module (GBP1, GBP2, GBP4, GBP5, STAT1, TAP1, CXCL10, IFIT3, EPSTI1, LAP3, and PARP14). SC-M4 versus SC-M2 differences were summarized using Cliff’s δ [33], and aggregate MT-ND and IFNγ-associated transcript shares were compared across AMP cell states. Cell-level effect sizes were interpreted descriptively because cells from the same donor are not independent biological replicates. Processed expression data and predefined myeloid clusters from the AMP Phase II single-cell analysis portal were additionally used to examine STAT1, C3, CD274, and IFIT1 expression across RA and OA myeloid populations [34]. The portal includes 73 RA samples and 9 OA samples; the RA sample count includes three repeated specimens and therefore does not represent 73 patients. The displayed gene-expression values were obtained directly from the portal and were not subjected to a new donor-level statistical analysis. Because the raw single-cell data were not reprocessed, the repeated specimens could not be incorporated into an independent-donor statistical model. These comparisons were therefore interpreted as exploratory and descriptive.

### 2.5. Pre-Ranked GSEA with clusterProfiler

For the comparative analysis of gene expression data across experimental groups, microarray probe identifiers were mapped to human gene symbols using Human_AFFY_HG_U95 for GSE1919, Human_AFFY_HG_U133A for GSE55457, and Human_AFFY_HG_U133_Plus_2 for GSE97779, corresponding to each dataset’s array platform (MSigDB v2024.1.Hs.Chip). This mapping ensured consistency across datasets, enabling accurate pathway enrichment analysis. Pre-ranked Gene Set Enrichment Analysis (GSEA) was performed using R (version 4.3.3) with the clusterProfiler package (version 4.10.1) to identify pathways relevant to biological processes, including inflammation, mitochondrial function, and immune response. We utilized the Hallmark and C2 gene set collections from the Molecular Signatures Database (MSigDB) for this analysis. Gene expression data were ranked by log fold change for each comparison group, and GSEA was executed with a maximum gene set size (MaxGSSize) of 1000, and a minimum gene set size (MinGSSize) of 10. Pathways were deemed significantly enriched if they met a *q*-value threshold of <0.05, which represents a false discovery rate (FDR)-corrected *p*-value, controlling for multiple testing.

### 2.6. Data Visualization with R and Python

Data visualization was carried out using ggplot2 (version 3.5.1) and forcats (version 1.0.0) in R (version 4.3.3), together with GSEApy (version 1.1.9) in Python (version 3.12.6). For enrichment dot plots generated in R, dot size represents statistical significance expressed as −log10(FDR *q*-value), while GeneRatio indicates the proportion of enriched genes relative to the pathway size. Graphical parameters for other dot plots are specified separately in the corresponding figure legends. Enrichment plots were generated to display the enrichment score (ES) across the ranked gene list, with the *x*-axis representing gene rank and the *y*-axis showing the ES. This visualization approach facilitated the identification and comparison of key pathways involved in inflammatory and mitochondrial functions across experimental groups.

## 3. Results

### 3.1. Distinct Hallmark Gene Set Enrichments in Synovial Tissue Between RA and OA Patients

**Figure 1:**
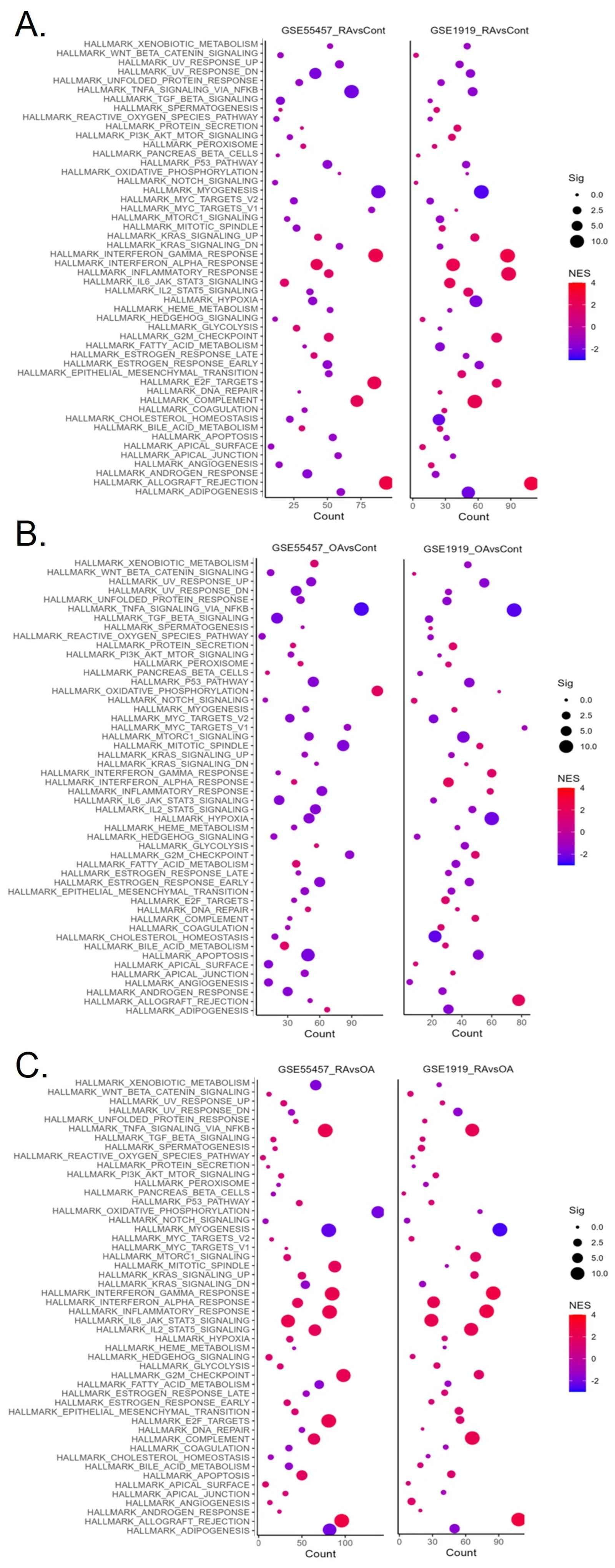
Hallmark pathway enrichment analysis of synovial tissue gene expression across arthritis conditions. Dot plots in (**A**–**C**) show hallmark pathway enrichment for RA vs. HC, OA vs. HC, and RA vs. OA comparisons, respectively, based on analyses of two independent clinical cohorts for each group. The *x*-axis indicates the number of significantly enriched genes within each pathway identified by the GSEA algorithm, and the *y*-axis displays 50 hallmark pathways. Dot color represents the normalized enrichment score (NES) (red, positive; blue, negative), and dot size reflects statistical significance expressed as −log10(FDR *q*-value).

To investigate the molecular signatures distinguishing RA from OA, we analyzed hallmark pathway enrichment in synovial tissue. In Figure 1, dot plots (Figure 1A–C) illustrate hallmark gene set enrichment in RA vs. healthy controls (HC), OA vs. HC, and RA vs. OA, respectively, while ridge plots (Supplementary Figure S1A–C) show the most significantly altered gene sets in each comparison. For RA synovial tissue compared to HC (Figure 1A), the IFNγ response gene set was prominently elevated, identifying a key inflammatory signature characteristic of RA. In parallel with the IFNγ response, additional immune-related pathways, including allograft rejection and complement activation, were consistently enriched across two independent cohort datasets (Supplementary Figure S1A).

In contrast, the OA vs. HC comparison (Figure 1B) revealed an overall attenuation of hallmark inflammatory pathways. Among these, TNFα signaling via NF-κB represented the most significantly reduced pathway, demonstrating a robust and reproducible decrease across two independent cohort datasets (Supplementary Figure S1B). The RA vs. OA comparison (Figure 1C) further highlighted distinct inflammatory signatures between the two conditions. Compared with OA, RA exhibited markedly stronger enrichment of inflammatory pathways, including IFNα and IFNγ responses, TNFα signaling via NF-κB, and IL-6/JAK/STAT3 and IL-2/STAT5 signaling. Consistent with the RA vs. HC comparison, the IFNγ response emerged as the most positively enriched signaling pathway in RA, whereas myogenesis represented the most negatively enriched pathway (Supplementary Figure S1C). Interestingly, although TNFα signaling via NF-κB was elevated in RA relative to OA, this pathway showed an overall reduction in the RA vs. HC comparison. Similarly, IL-6/JAK/STAT3 and IL-2/STAT5 signaling pathways were less prominently enriched than the IFNα and IFNγ responses.

### 3.2. Synovial Fluid-Derived RA Macrophages Reflect Inflammatory Pathways Observed in RA Synovial Tissue

**Figure 2:**
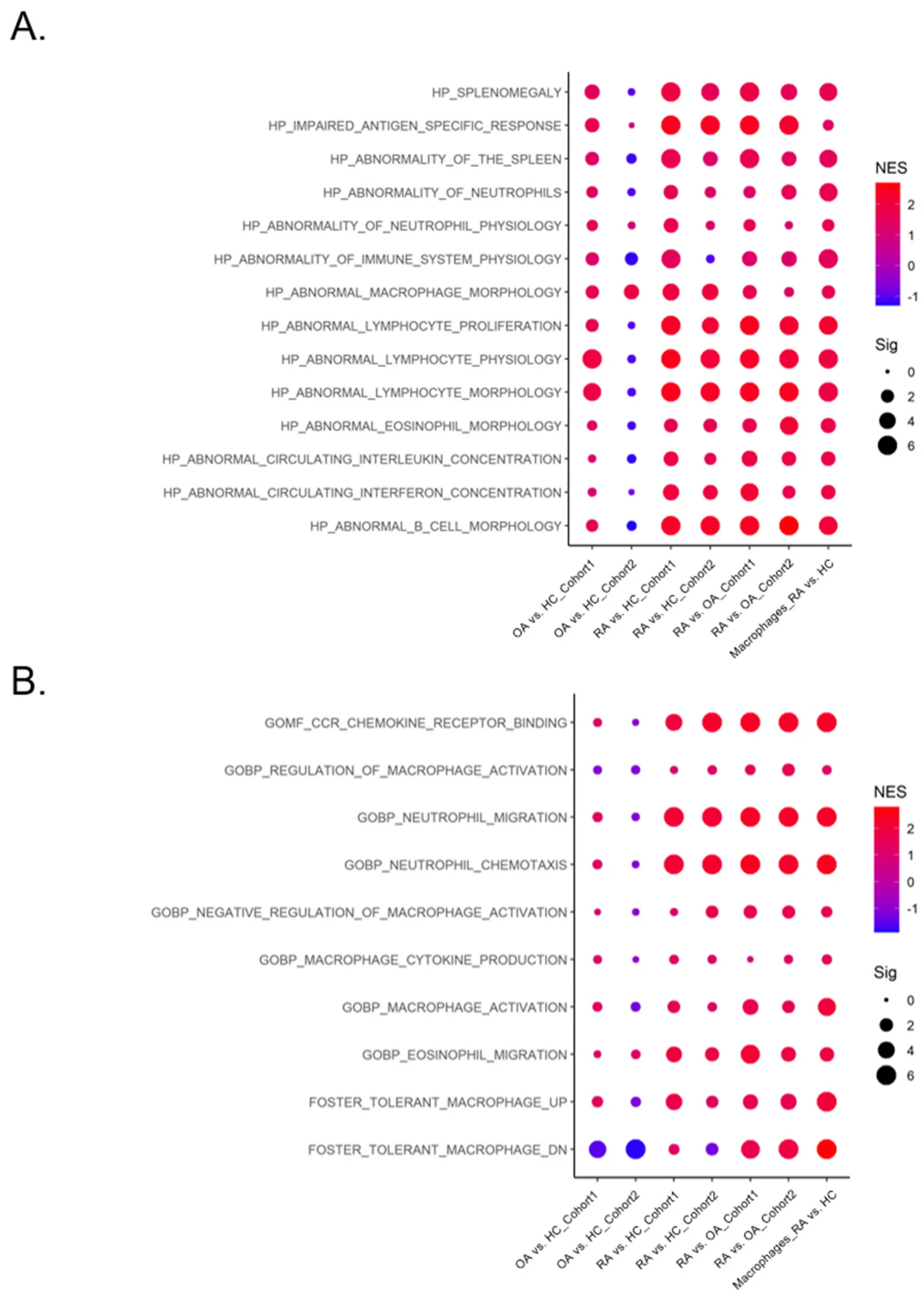

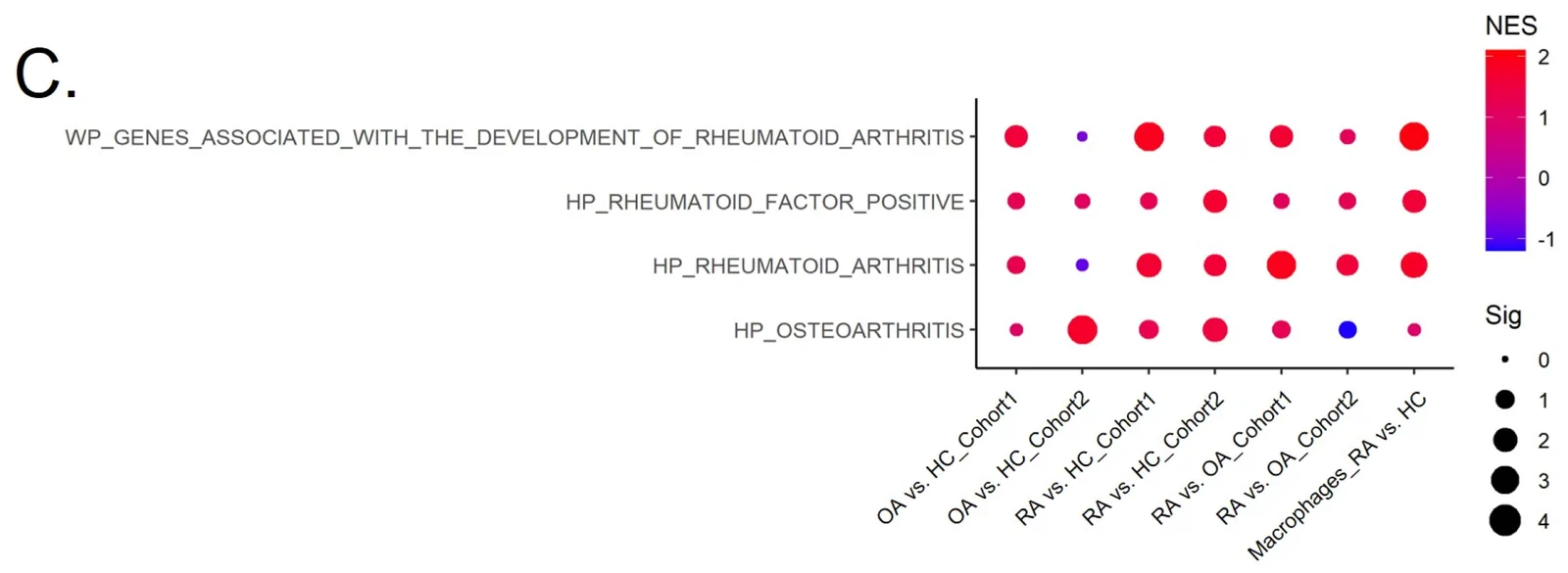
Comparative enrichment analysis of immune responses in synovial tissue Cohorts 1 and 2 and synovial fluid-derived RA macrophages across arthritis conditions. (**A**) A dot plot displays gene set enrichment associated with abnormal immune responses in synovial tissue Cohorts 1 and 2 (RA vs. HC, OA vs. HC, and RA vs. OA) and synovial fluid-derived RA macrophages (RA vs. HC). Dot color indicates the NES (red for positive enrichment, blue for negative enrichment), and dot size represents the significance level, calculated as −log10(FDR *q*-value). (**B**) A dot plot illustrates gene sets involved in innate immune responses in synovial tissue Cohorts 1 and 2 and synovial fluid-derived RA macrophages. (**C**) A dot plot presents gene sets associated with rheumatoid arthritis development, rheumatoid factor positivity, and osteoarthritis, distinguishing RA- and OA-specific enrichment patterns. Cohort 1 corresponds to GSE55457, and Cohort 2 corresponds to GSE1919.

To determine whether the tissue-level inflammatory and metabolic signatures observed in RA synovium are reflected at the cellular level, we next compared pathway enrichment profiles between synovial fluid-derived RA macrophages (RA vs. HC) and synovial tissue (RA vs. OA) across relevant comparison groups in Figure 2. Synovial fluid-derived RA macrophages consistently exhibited RA-specific immune changes, particularly in pathways associated with altered cell morphology and immune function, with significant enrichment in macrophage and neutrophil activation observed in both RA synovial tissue and synovial fluid-derived RA macrophages (Figure 2A). These shared patterns indicate overlap between transcriptional programs in synovial fluid-derived RA macrophages and those observed in RA tissue.

Consistently, Figure 2B further demonstrates that synovial fluid-derived RA macrophages recapitulated several tissue-level immune alterations, particularly in pathways related to macrophage activation, neutrophil recruitment, and immune tolerance. Gene sets related to macrophage tolerance showed both positive and negative enrichment, suggesting heterogeneous regulation of tolerance-associated transcriptional programs rather than a uniformly activated or suppressed state. Additionally, pathways linked to arthritis-specific immune responses, including rheumatoid factor positivity and RA autoimmunity (Figure 2C), were enriched, suggesting that macrophages reflect not only general inflammation but also RA-associated immune transcriptional programs.

### 3.3. Mitochondrial Complex I-Related Transcriptional Suppression Is Prominent in RA Synovium and Synovial Fluid-Derived RA Macrophages

**Figure 3:**
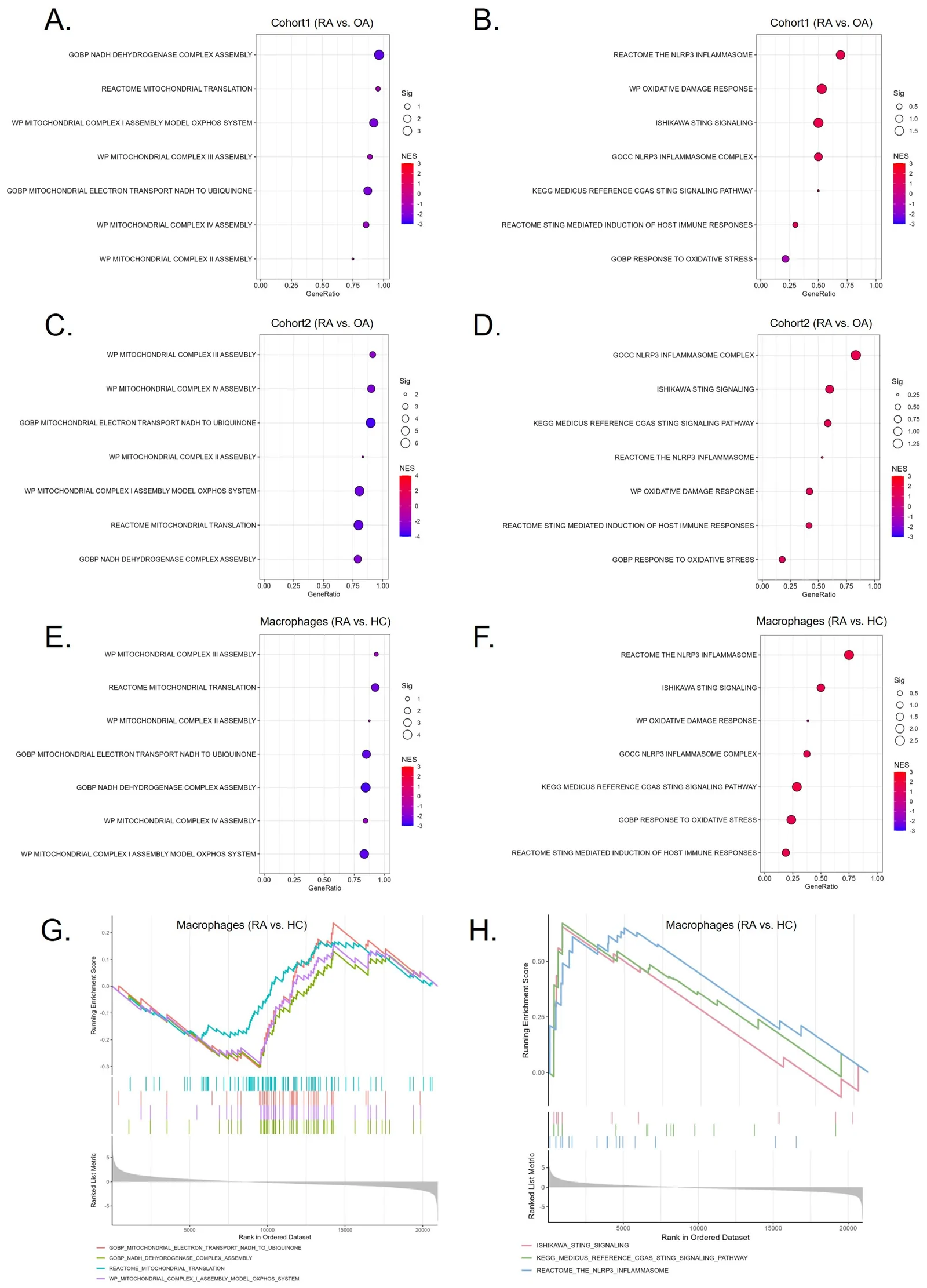
Comparative enrichment analysis of mitochondrial function in synovial tissue and synovial fluid-derived RA macrophages across arthritis conditions. (**A**,**B**) Dot plots display gene set enrichment associated with mitochondrial bioenergetics (**A**) and NLRP3 inflammatory responses (**B**) in synovial tissue Cohort 1 (RA vs. OA). (**C**,**D**) Dot plots illustrate gene set enrichment associated with mitochondrial bioenergetics (**C**) and NLRP3 inflammatory responses (**D**) in synovial tissue Cohort 2 (RA vs. OA). (**E**,**F**) Dot plots show gene set enrichment associated with mitochondrial bioenergetics (**E**) and NLRP3 inflammatory responses (**F**) in synovial fluid-derived RA macrophages versus M-CSF-derived control macrophages (GSE97779; RA vs. HC). The *x*-axis denotes the gene ratio, indicating the proportion of pathway-related genes with significant enrichment. Gene set names are listed on the *y*-axis, with dot color indicating the NES and dot size representing the significance level, calculated as −log10(FDR *q*-value). (**G**,**H**) Representative GSEA graphs illustrating enrichment patterns of gene sets associated with CI activity (**G**) and NLRP3 inflammasome activation (**H**) in synovial fluid-derived RA macrophages (RA vs. HC).

Given that macrophages are central contributors to inflammatory processes in RA, we next examined mitochondrial transcriptional programs, with particular focus on the electron transport chain. Across the analyzed comparisons, gene sets related to mitochondrial Complex I (CI) showed more prominent negative enrichment than those related to complexes II–IV (Figure 3A,C,E). This pattern was also observed in synovial fluid-derived RA macrophages, supporting an association between RA inflammatory macrophage states and reduced CI-related transcription.

Mitochondrial translation-related gene sets were concurrently suppressed with CI-related pathways, indicating coordinated alteration of mitochondrial transcriptional programs. Because mitochondrial translation is required for synthesis of mtDNA-encoded respiratory-chain subunits, including the seven MT-ND components of CI, this pattern is consistent with reduced capacity for CI biogenesis, although CI enzymatic function was not directly measured in these datasets.

Because mitochondrial perturbation has been linked to mtDNA-dependent innate immune signaling, we additionally examined cGAS-STING- and NLRP3-related gene sets. These pathways showed positive enrichment across the analyzed comparisons, although the magnitude and statistical significance were modest (Figure 3B,D,F). Accordingly, these results support an association between reduced CI-related transcription and increased innate immune signaling signatures, rather than establishing a sequential pathway from CI impairment to cGAS-STING or NLRP3 activation. Figure 3G,H further illustrate GSEA enrichment patterns for CI-related pathways and the NLRP3 inflammasome signature in synovial fluid-derived RA macrophages, respectively.

### 3.4. Distinct Immunometabolic Patterns in Synovial Fluid-Derived RA and Synovial Tissue-Derived OA Macrophages

Based on the preceding findings, the metabolic phenotype observed in synovial fluid-derived RA macrophages may not represent a simple shift toward classical M1 polarization but rather a transcriptional state characterized by reduced OXPHOS- and Complex I-related programs. To further investigate this, we performed a more detailed analysis of immunometabolic alterations in synovial fluid-derived RA macrophages and independently examined metabolic patterns in synovial tissue-derived inflammatory OA macrophages.

**Figure 4:**
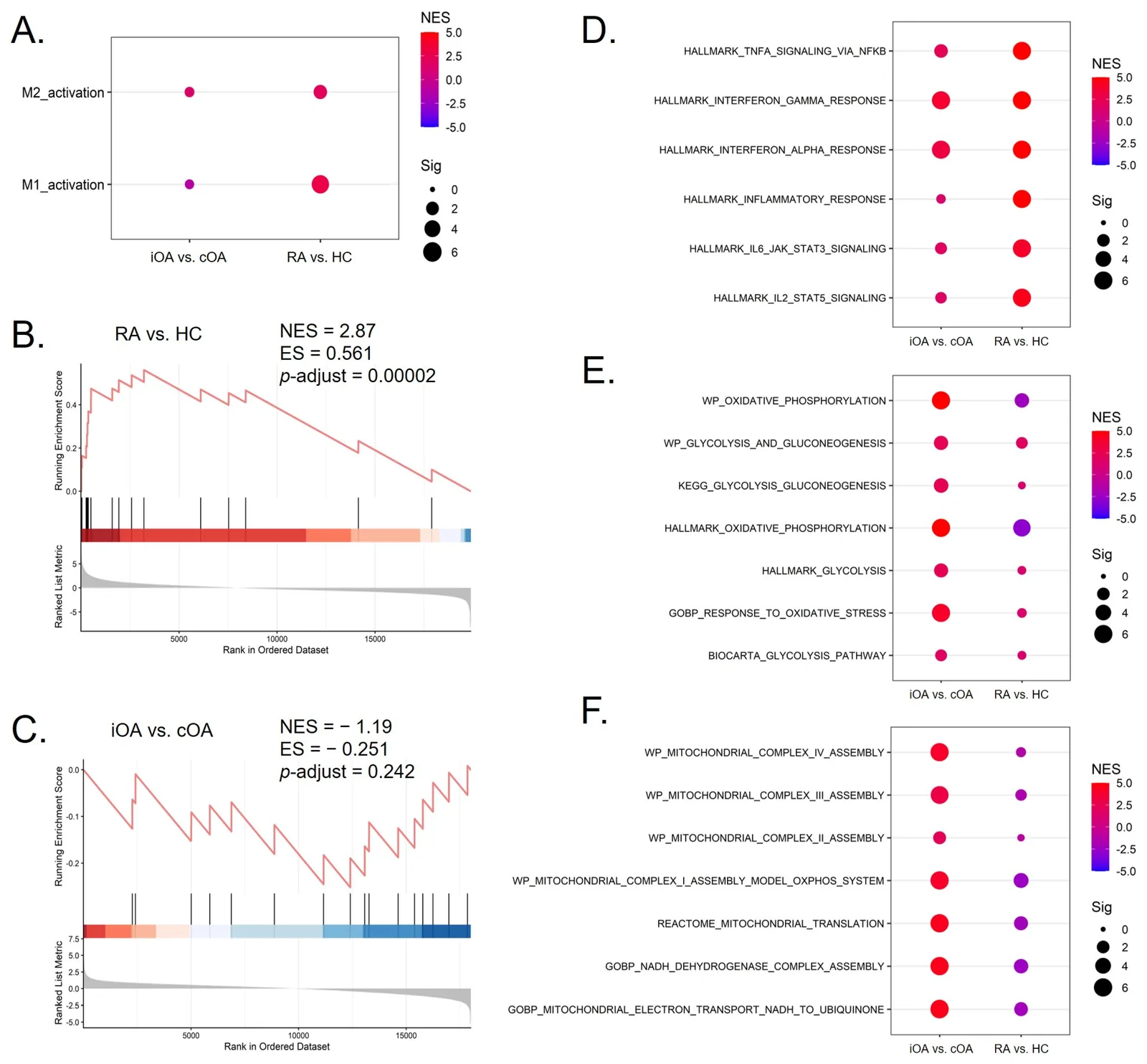
Distinct inflammatory and metabolic pathway signatures in synovial fluid-derived RA and synovial tissue-derived OA macrophages. (**A**) Dot plot summarizing enrichment analysis results for M1 and M2 macrophage activation signatures across disease comparisons (iOA vs. cOA and RA vs. HC). Color represents the NES and size indicates statistical significance, calculated as −log10(FDR *q*-value). (**B**,**C**) Enrichment plots of the M1 activation gene set across disease comparisons: RA vs. HC (**B**) and iOA vs. cOA (**C**). The *y*-axis represents the running enrichment score (ES), and the *x*-axis represents the rank in the ordered gene list. The red-to-blue color gradient represents the ranked-list metric, ranging from positive to negative values, and the gray distribution at the bottom shows the magnitude and direction of the ranked-list metric. (**D**–**F**) Dot plots showing enrichment analysis results for inflammatory signaling pathways (**D**), metabolic pathways (**E**), and mitochondrial respiratory chain and complex assembly pathways (**F**) across disease comparisons. Dot color indicates the NES, and dot size represents the significance level, calculated as −log10(FDR *q*-value).

In RA versus HC (Figure 4A,B), both M1- and M2-associated gene sets were elevated; however, M1-associated pathways showed stronger enrichment, which was further supported by the positive enrichment of M1-related gene sets (Figure 4B). Consistent with this, metabolic profiling showed reduced OXPHOS-related transcriptional programs. However, glycolysis did not exhibit a corresponding significant increase, indicating that the metabolic state of RA macrophages does not fully conform to a canonical M1-like glycolytic shift. In addition, the concurrent suppression of mitochondrial translation and Complex I-related gene sets supports a transcriptionally constrained mitochondrial program involving Complex I rather than directly demonstrating impaired OXPHOS function.

Conversely, comparison of synovial tissue-derived macrophages between iOA and cOA patients revealed no significant increase in M1-associated gene sets (Figure 4C). In contrast to the RA macrophage dataset, synovial tissue-derived macrophages from iOA patients exhibited concurrent enrichment of OXPHOS, glycolysis, and ETC-related transcriptional programs (Figure 4D–F), indicating a metabolically active inflammatory state within this OA subgroup. Because the RA macrophages were isolated from synovial fluid, whereas the OA macrophages were isolated from synovial tissue, and the analyses were derived from independent published datasets with different comparator groups, these findings were not interpreted as a direct statistical comparison between RA and OA macrophages. We therefore further examined RA and OA synovial monocytes within a common study framework.

### 3.5. IFN-Responsive Inflammatory Monocytes Show Reduced Mitochondrial Translation and Complex I-Related Transcription in RA

**Figure 5:**
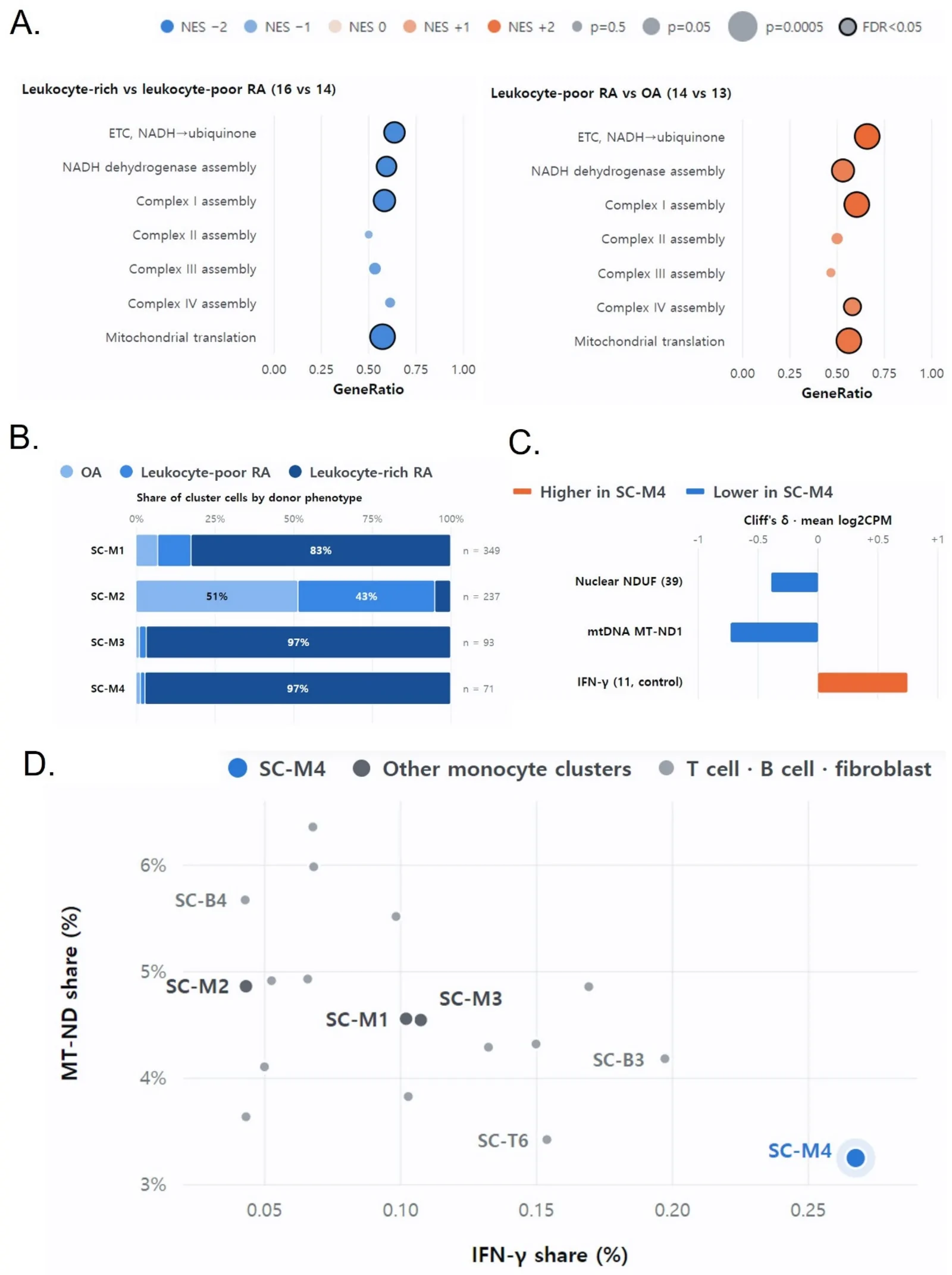
Bulk and single-cell transcriptomic analysis of mitochondrial Complex I and IFN-responsive programs in RA and OA synovial monocytes. (**A**) Pre-ranked GSEA of sorted synovial monocyte bulk RNA-seq from the AMP Phase I cohort comparing leukocyte-rich versus leukocyte-poor RA (*n* = 16 vs. 14) and leukocyte-poor RA versus OA (*n* = 14 vs. 13), using the same predefined mitochondrial gene sets applied in the preceding analyses. Dot color indicates NES, dot size indicates permutation-derived *p*-value, and outlined dots indicate FDR < 0.05. (**B**) Distribution of the previously defined monocyte states SC-M1-SC-M4 across OA, leukocyte-poor RA, and leukocyte-rich RA phenotypes. (**C**) Cliff’s δ effect sizes for SC-M4 versus SC-M2 based on the 39-gene nuclear NDUF set, MT-ND1, and the 11-gene IFN-γ- associated transcriptional module. Negative values indicate lower expression and positive values indicate higher expression in SC-M4. (**D**) Relationship between the aggregate transcript share of the 11-gene IFNγ-associated module and that of the seven mtDNA-encoded Complex I genes (MT-ND1, MT-ND2, MT-ND3, MT-ND4, MT-ND4L, MT-ND5, and MT-ND6) across AMP cell states.

To examine RA and OA synovial monocytes within a common study framework, we reanalyzed AMP Phase I bulk and single-cell RNA-seq data. In sorted monocytes, leukocyte-rich RA showed negative enrichment of multiple mitochondrial pathways relative to leukocyte-poor RA. Using the same predefined mitochondrial gene sets as in the preceding analyses, reduced enrichment was observed for NADH-to-ubiquinone electron transport, NADH dehydrogenase and Complex I assembly, and mitochondrial translation (Figure 5A). We further performed a direct comparison between leukocyte-rich RA and OA using a predefined gene set representative of mitochondrial Complex I assembly (Supplementary Figure S3). This comparison showed a pattern similar to that observed between leukocyte-rich and leukocyte-poor RA, with reduced expression of Complex I assembly-related genes in leukocyte-rich RA. Although leukocyte-poor RA versus OA also showed statistically significant enrichment, the corresponding gene-level changes were generally smaller. Gene-level effect sizes, leading-edge genes, and sample-level expression patterns further demonstrated the direction and magnitude of these changes, as well as inter-sample variability across the three groups (Supplementary Figure S3). Collectively, these findings indicate that suppression of mitochondrial translation and Complex I-related transcription is preferentially associated with the leukocyte-rich inflammatory RA state. Complete enrichment statistics and leading-edge genes are provided in Supplementary Table S2.

At single-cell resolution, 97% of SC-M4 cells originated from leukocyte-rich RA donors, whereas SC-M2 was predominantly derived from OA and leukocyte-poor RA donors (Figure 5B). Compared with SC-M2, SC-M4 showed lower expression of the 39-gene nuclear NDUF set (Supplementary Table S1) and MT-ND1, together with markedly higher expression of the 11-gene IFNγ-associated transcriptional module (GBP1, GBP2, GBP4, GBP5, STAT1, TAP1, CXCL10, IFIT3, EPSTI1, LAP3, and PARP14) (Figure 5C).

Consistently, across AMP cell states, SC-M4 displayed the highest IFNγ-response transcript share and the lowest aggregate share of the seven mtDNA-encoded Complex I genes (MT-ND1, MT-ND2, MT-ND3, MT-ND4, MT-ND4L, MT-ND5, and MT-ND6) among monocyte populations (Figure 5D). Together, these findings associate the IFN-responsive SC-M4 state with reduced nuclear and mtDNA-encoded Complex I-related transcription and link this state to the suppressed mitochondrial translation program observed in leukocyte-rich RA. Although compatible with prominent IFNγ-associated signaling in the bulk analyses, the SC-M4 signature was not considered specific to type II interferon and may reflect convergent type I and type II interferon responses.

### 3.6. STAT1–CXCL10-High Macrophages Exhibit Differential Interferon-Responsive Features in RA and OA

**Figure 6:**
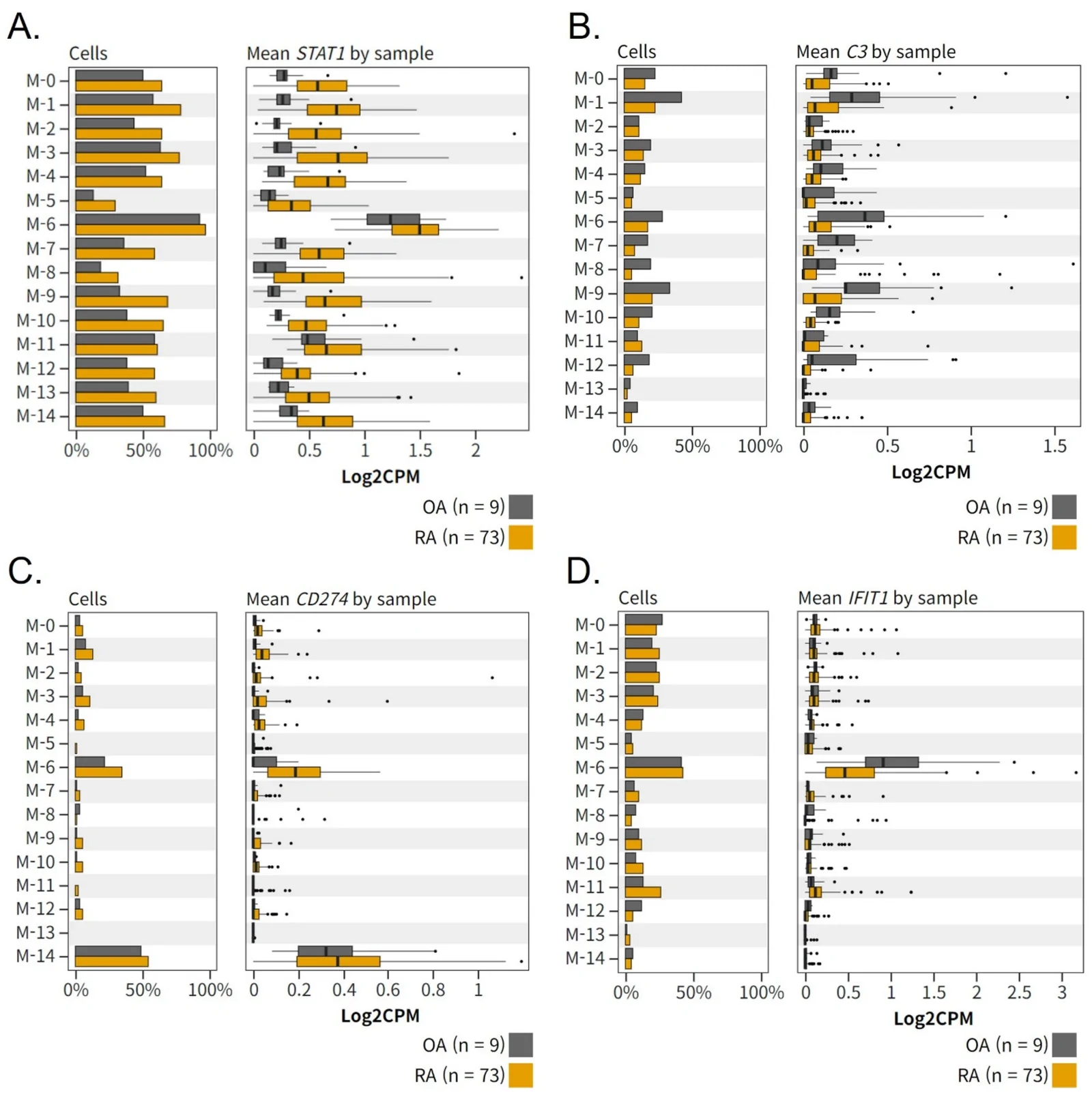
Comparison of synovial tissue-derived myeloid cells between RA and OA using the AMP Phase II scRNA-seq dataset. Expression of STAT1 (**A**), C3 (**B**), CD274 (**C**), and IFIT1 (**D**) across myeloid clusters. Bar plots show the percentage of cells expressing each gene within a cluster. Box plots summarize sample-level mean log2CPM values for RA (*n* = 73, including three repeated specimens) and OA (*n* = 9). Boxes indicate the interquartile range, vertical black lines indicate the median, horizontal lines extend to the most extreme values within 1.5 times the interquartile range, and black dots indicate outliers. OA and RA are shown in gray and orange, respectively. Data were obtained from the publicly available AMP Phase II portal developed by Zhang et al. (2023) [34].

Building on the association between IFN-responsive monocyte states and reduced Complex I-related transcription, we next examined IFN-responsive synovial tissue-derived macrophage states in RA and OA using single-cell data from the AMP Phase II analysis portal [34] (Figure 6 and Supplementary Figure S2). The previously defined STAT1–CXCL10-high macrophage population (M6) was present in both RA and OA and exhibited a prominent IFN-responsive transcriptional profile (Figure 6A and Supplementary Figure S2).

Within this population, RA macrophages showed relatively increased expression of the immune checkpoint molecule CD274 and reduced expression of the IFN-stimulated gene IFIT1 compared with OA macrophages (Figure 6C,D). These differences suggest altered regulation of IFN-responsive transcription in RA rather than a uniformly enhanced acute interferon response. C3 expression was comparatively higher in OA macrophages (Figure 6B), further indicating disease-associated differences within this IFN-responsive macrophage state.

Collectively, comparison with the corresponding STAT1–CXCL10-high macrophage population in OA highlighted a relatively restrained IFN-responsive pattern in RA. Although this population was present in both diseases, RA macrophages showed relatively lower IFIT1 despite maintained STAT1 expression, together with higher CD274.

## 4. Discussion

In this study, we found that synovial fluid-derived RA macrophages exhibit a distinct immunometabolic phenotype characterized by suppression of CI-associated pathways, in association with persistent IFNγ-associated signaling. In an independent dataset of synovial tissue-derived OA macrophages, inflammatory OA macrophages showed concurrent activation of OXPHOS and glycolysis, whereas synovial fluid-derived RA macrophages showed reduced OXPHOS-related transcription without a corresponding increase in glycolysis. Because these macrophage analyses differed in anatomical source and were derived from independent datasets with different comparator groups, they were not interpreted as a direct RA–OA metabolic comparison.

The inflammatory features observed in RA appeared to reflect a qualitatively distinct immune state rather than simply stronger inflammation compared with OA. In our study, IFNγ-associated signaling represented the most prominently enriched inflammatory pathway in RA compared with OA. As discussed above, the role of IFNγ in RA, similar to other autoimmune diseases, appears to exhibit dual-edged effects. In this context, persistent interferon activation may paradoxically contribute to dysregulated inflammatory responses rather than sustained classical inflammatory amplification. Consistent with this possibility, TNFα signaling via NF-κB and NLRP3 inflammasome-related pathways were not proportionally elevated despite the strong IFN-associated signatures observed in RA. Although TNFα signaling via NF-κB was relatively increased in RA compared with OA, its overall pathway enrichment in RA tended to remain lower than that observed in HC. This reduction may reflect intrinsic negative feedback regulation of the NF-κB pathway under chronic inflammatory conditions. The different TNFα signaling patterns observed across comparisons may reflect context-dependent regulation of inflammatory pathways in RA and OA. These differences may partly explain the distinct effects of TNFα inhibitors observed between RA and OA [35,36] and may also contribute to the context-dependent effects of TNFα signaling in RA [37].

Consistent with the reduced enrichment of the TNFα signaling via NF-κB, NLRP3 inflammasome-associated pathways in RA exhibited only modest enrichment. This suggests that rather than acting as a primary driver, the NLRP3 axis may function as a secondary factor or a context-specific mediator in maintaining chronic RA inflammation [38]. The modest NLRP3-associated enrichment supports a potential association with chronic RA inflammation but does not establish a causal link between CI suppression and NLRP3 activation.

IFNγ, essential for inducing Th1 responses, requires mitochondrial CI activity to sustain energy production and ROS generation, both of which are critical for Th1 activation. However, once activated, Th1 cells downregulate mitochondrial CI activity, along with other components of the ETC and OXPHOS, in favor of glycolysis. This metabolic rewiring enhances pro-inflammatory responses in effector T cells, as it prevents LDH (lactate dehydrogenase) from binding to the untranslated regions (UTRs) of pro-inflammatory mRNAs, such as IFNγ and TNFα, thereby amplifying their expression [39]. Chronic expression of IFNγ, however, can lead to dysregulation of mitochondrial CI activity, potentially disrupting overall ETC function [40,41]. Consistent with this possibility, our bulk and single-cell RNA-seq analysis showed reduced mitochondrial translation and CI-related programs in leukocyte-rich RA monocytes. IFN-responsive monocytes additionally showed reduced nuclear- and mtDNA-encoded CI-related transcription. Because the seven mtDNA-encoded MT-ND subunits require mitochondrial translation for protein synthesis [42], these findings support a model in which persistent IFNγ-associated signaling may compromise CI biogenesis and increase susceptibility to CI dysfunction.

To further characterize regulatory features within the IFN-responsive macrophage population, we focused on macrophages with high STAT1 and CXCL10 expression. This population showed lower C3 and IFIT1 expression in RA than in OA, together with relatively higher CD274 expression, consistent with altered regulatory features of chronic IFNγ-responsive transcription [43]. Consistent with this interpretation, a recent review proposed that chronic IFNγ exposure can induce adaptive regulatory programs in macrophages, including checkpoint induction, suppressive feedback signaling, metabolic rewiring, and epigenetic remodeling [44]. Relatively higher C3 expression in OA may reflect differences in complement-associated responses between RA and OA macrophages. A preclinical study using human chondrocytes and a spontaneous OA guinea-pig model showed that complement C3 activation can contribute to OA-associated cartilage damage [45], indicating that the biological consequences of C3 signaling are likely context dependent. In RA, lower C3 expression together with increased CD274 and reduced IFIT1 may reflect altered regulation of the STAT1–CXCL10-high IFN-responsive macrophage state. However, these transcriptional differences alone are insufficient to establish secondary exhaustion or defective inflammatory resolution.

Several limitations should be considered. First, this study integrates publicly available datasets with relatively small and unequal sample sizes, and detailed clinical information, including treatment status and OA phenotype, was not consistently available across cohorts. Second, the RA macrophages in GSE97779 were isolated from synovial fluid, whereas the OA macrophages in GSE123492 were isolated from synovial tissue. These differences in anatomical source, cell preparation, assay platform, and comparator groups limit direct comparison between the independent RA and OA macrophage datasets. The transcriptionally constrained mitochondrial phenotype observed here should not be generalized to all RA macrophages, as metabolic phenotypes may vary according to cell origin, microenvironment, disease stage, and macrophage state. Further single-cell comparisons across disease stages and treatment outcomes, including remission and treatment resistance, will help define this heterogeneity. Third, the present findings are primarily transcriptomic and do not directly establish mitochondrial respiratory function, Complex I enzymatic activity, metabolic flux, or causal effects of IFNγ signaling. Future studies using standardized patient cohorts with detailed clinical annotation and orthogonal functional validation are therefore warranted.

In summary, our study identifies prominent IFNγ-associated signaling together with reduced mitochondrial translation and Complex I-related transcription in inflammatory RA synovial myeloid cells. In particular, IFN-responsive monocytes showed reduced transcriptional representation of both nuclear- and mtDNA-encoded Complex I components. These findings identify an association between an IFN-responsive inflammatory myeloid state and reduced mitochondrial translation and Complex I-related transcription in RA, providing a framework for future functional studies of immunometabolic heterogeneity in inflammatory synovitis.

## Data Availability

All datasets analyzed in this study are publicly available and were obtained from previously published sources. Detailed information on dataset accession numbers and sources is provided in Section 2. Additional data and analysis code supporting the findings of this study are available from the corresponding author upon reasonable request.

## Supplementary Materials

The following supporting information can be downloaded at: https://www.mdpi.com/article/10.3390/biomedicines14092094/s1, Figure S1: Top statistically significant Hallmark pathways from synovial tissue gene expression in RA and OA patients; Figure S2: Single-cell characterization of synovial myeloid cells from patients with RA and OA in AMP Phase II dataset; Figure S3: NADH dehydrogenase complex assembly enrichment in synovial monocytes; Table S1: 39-gene nuclear NDUF set; Table S2: Gene set enrichment results and leading-edge genes.

## Abbreviations

RA	Rheumatoid arthritis
OA	Osteoarthritis
HC	Healthy control
IFNγ	Interferon-gamma
TNFα	Tumor necrosis factor-alpha
IL	Interleukin
JAK	Janus kinase
OXPHOS	Oxidative phosphorylation
ROS	Reactive oxygen species
mtDNA	Mitochondrial DNA
cGAS	Cyclic GMP-AMP synthase
STING	Stimulator of interferon genes
NLRP3	NOD-like receptor family pyrin domain containing 3
CI	Mitochondrial complex I
ETC	Electron transport chain
GSEA	Gene set enrichment analysis
NES	Normalized enrichment score
FDR	False discovery rate
GEO	Gene Expression Omnibus
scRNA-seq	Single-cell RNA sequencing
iOA	Inflammatory-like osteoarthritis
cOA	Conventional osteoarthritis
ES	Enrichment score
PKR	Protein kinase R
