# Supplementary material for: Patient-Derived Transcriptomics Reveals Distinct IFNγ-Associated Myeloid States and Mitochondrial Complex I Programs in Rheumatoid Arthritis and Osteoarthritis
Source: Biomedicines. 2026 Sep 17;14(9):2094. doi: 10.3390/biomedicines14092094 (PMC13604131; doi:10.3390/biomedicines14092094)
Supplement: Supplementary file 1 [file biomedicines-14-02094-s001.zip › biomedicines-4510548-supplementary.pdf]

Supplementary Figure S1.

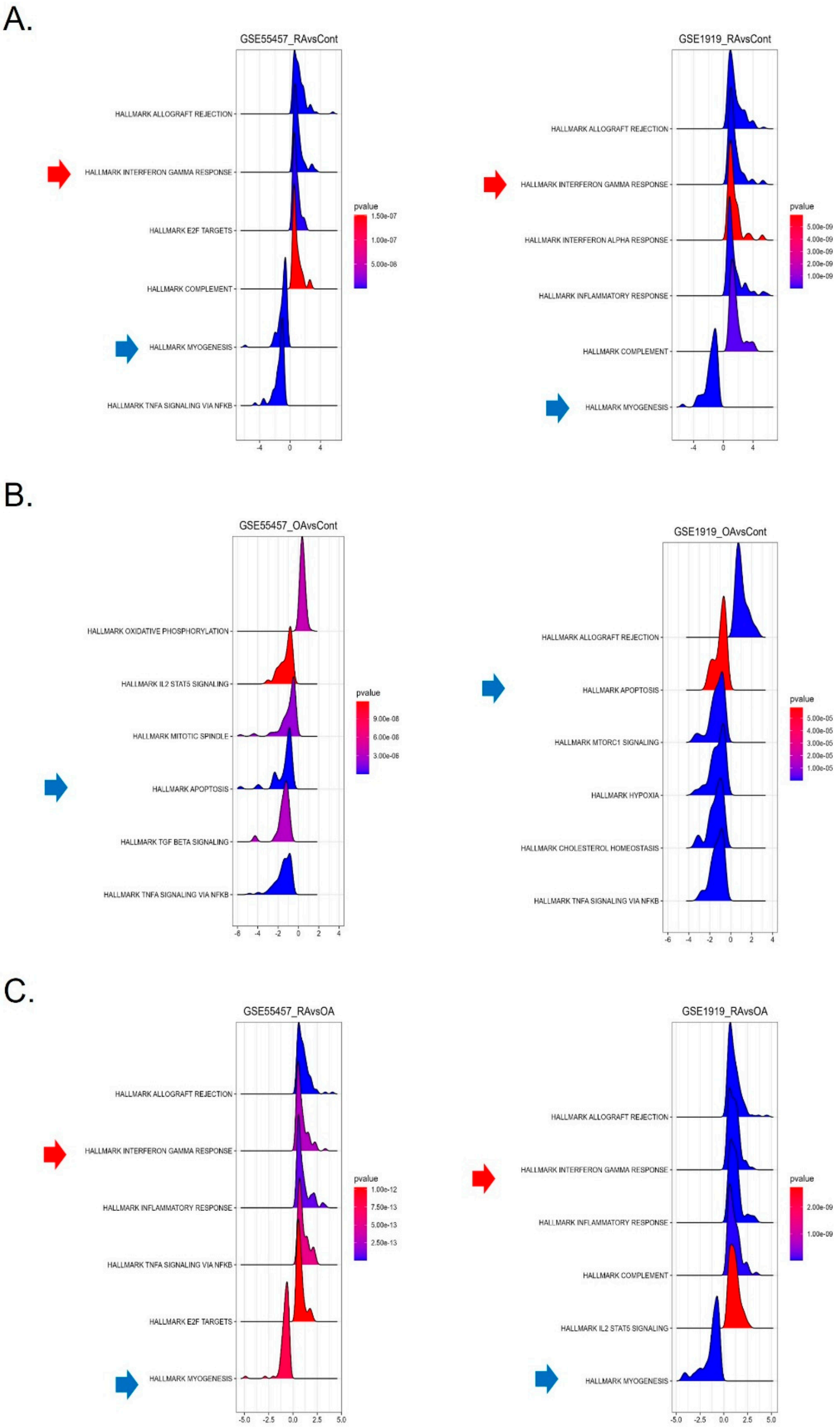

Supplementary Figure S1. Top statistically significant Hallmark pathways from synovial tissue gene expression in RA and OA patients. Ridge plots of (A), (B), and (C) present the top enriched pathways from figure 1A. The x-axis displays ranges of NES, while the y-axis lists the top upregulated and downregulated pathways based on the absolute value of NES. Pathways indicated by red arrows show consistent upregulation, while blue arrows denote consistent downregulation.

Supplementary Figure S2.

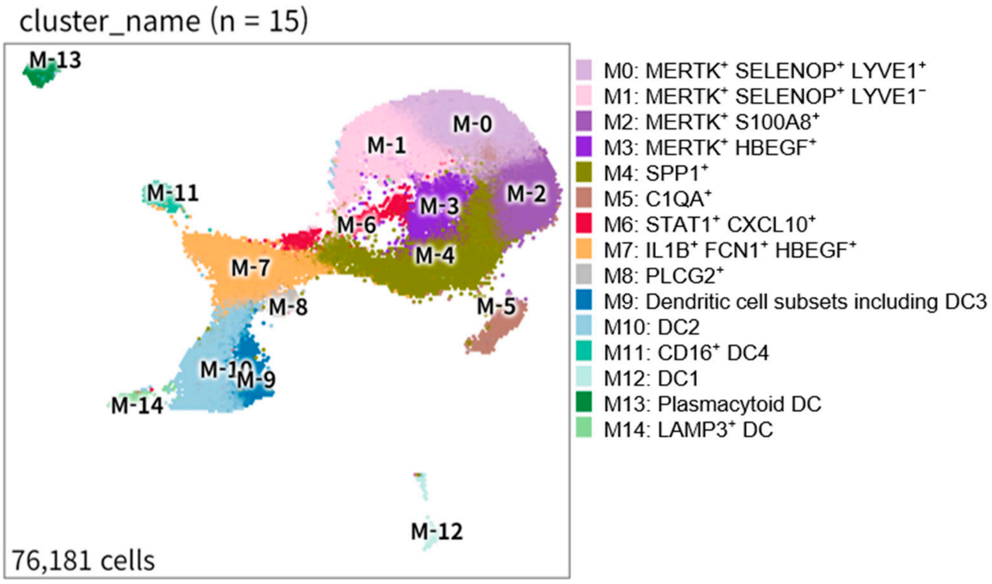

Supplementary Figure S2. Single-cell characterization of synovial myeloid cells from patients with rheumatoid arthritis and osteoarthritis. Two-dimensional embedding of 76,181 synovial myeloid cells from patients with rheumatoid arthritis (RA) and osteoarthritis (OA), showing 15 distinct clusters (M0–M14).

Supplementary Table S1.

39-gene nuclear NDUF set

1	NDUFS1	Core N/Q module
2	NDUFS2	Core N/Q module
3	NDUFS3	Core N/Q module
4	NDUFS7	Core N/Q module
5	NDUFS8	Core N/Q module
6	NDUFV1	Core N/Q module
7	NDUFV2	Core N/Q module
8	NDUFA1	Accessory NDUFA
9	NDUFA2	Accessory NDUFA
10	NDUFA3	Accessory NDUFA
11	NDUFA5	Accessory NDUFA
12	NDUFA6	Accessory NDUFA
13	NDUFA8	Accessory NDUFA
14	NDUFA9	Accessory NDUFA
15	NDUFA10	Accessory NDUFA
16	NDUFA11	Accessory NDUFA
17	NDUFA12	Accessory NDUFA
18	NDUFA13	Accessory NDUFA
19	NDUFAB1	Accessory NDUFA
20	NDUFB1	Accessory NDUFB
21	NDUFB2	Accessory NDUFB
22	NDUFB3	Accessory NDUFB
23	NDUFB4	Accessory NDUFB
24	NDUFB5	Accessory NDUFB
25	NDUFB6	Accessory NDUFB
26	NDUFB7	Accessory NDUFB
27	NDUFB9	Accessory NDUFB
28	NDUFB10	Accessory NDUFB
29	NDUFB11	Accessory NDUFB
30	NDUFC1	Other NDUF
31	NDUFC2	Other NDUF
32	NDUFS4	Other NDUF
33	NDUFS5	Other NDUF
34	NDUFS6	Other NDUF
35	NDUFV3	Other NDUF
36	NDUFA4	NDUF-associated/non-canonical
37	NDUFA4L2	NDUF-associated/non-canonical
38	NDUFA6-AS1	NDUF-associated/non-canonical
39	NDUFB2-AS1	NDUF-associated/non-canonical

Supplementary Table S2.

Table S2a. Gene set enrichment results

Table S2a-1. Leukocyte-rich vs leukocyte-poor RA (16 vs 14 donors; bulk RNA-seq).

					Gene-set permutation		Sample-label permutation		
**Gene set**	**MSigDB**	**In set**	**In list**	**NES**	***Nominal P***	**FDR**	***Nominal P***	**FDR**	**Leading edge**
ETC, NADH to ubiquinone	M22379	53	44	**-1.763**	0.0020	**0.0058**	0.1519	0.2547	28 / 44
NADH dehydrogenase assembly	M22615	41	32	**-1.793**	0.0035	**0.0061**	0.0570	0.2547	19 / 32
Complex I assembly	M39781	56	48	**-1.745**	0.0025	**0.0058**	0.1349	0.2547	28 / 48
Complex II assembly	M39898	8	8	**-0.937**	0.3193	0.3193	0.3218	0.3218	4 / 8
Complex III assembly	M39892	16	15	**-1.357**	0.0765	0.1070	0.1819	0.2547	8 / 15
Complex IV assembly	M39881	34	31	**-1.208**	0.1359	0.1586	0.2894	0.3218	19 / 31
Mitochondrial translation	M27446	115	96	**-1.860**	<0.0005	**0.0035**	0.1339	0.2547	55 / 96

Table S2a-2. Leukocyte-poor RA vs OA (14 vs 13 donors; bulk RNA-seq).

					Gene-set permutation		Sample-label permutation		
**Gene set**	**MSigDB**	**In set**	**In list**	**NES**	***Nominal P***	**FDR**	***Nominal P***	**FDR**	**Leading edge**
ETC, NADH to ubiquinone	M22379	53	44	**+2.196**	<0.0005	**0.0012**	0.0245	0.1224	29 / 44
NADH dehydrogenase assembly	M22615	41	32	**+1.736**	0.0035	**0.0061**	0.0565	0.1318	17 / 32
Complex I assembly	M39781	56	48	**+2.133**	<0.0005	**0.0012**	0.0350	0.1224	29 / 48
Complex II assembly	M39898	8	8	**+1.340**	0.0810	0.0945	0.1304	0.1621	4 / 8
Complex III assembly	M39892	16	15	**+1.131**	0.2084	0.2084	0.2499	0.2499	7 / 15
Complex IV assembly	M39881	34	31	**+1.616**	0.0110	**0.0154**	0.1389	0.1621	18 / 31
Mitochondrial translation	M27446	115	96	**+1.969**	<0.0005	**0.0012**	0.1034	0.1621	54 / 96

Table S2b. Leading-edge genes

Table S2b-1. Leukocyte-rich vs leukocyte-poor RA.

**Gene set**	**n**	**Leading-edge genes**
ETC, NADH to ubiquinone	28	NDUFB6, NDUFA4, NDUFS3, ISCU, NDUFA8, SNCA, NDUFA4L2, NDUFC1, NDUFS5, NDUFV1, NDUFB2, NDUFB7, NDUFAB1, NDUFS2, NDUFA5, NDUFB10, NDUFB3, NDUFB4, NDUFB9, NDUFS6, NDUFS8, NDUFC2, NDUFA1, NDUFV2, DLD, NDUFS7, NDUFS1, NDUFA9
NADH dehydrogenase assembly	19	NDUFAF4, BCS1L, TMEM186, TIMM21, NDUFS3, TIMMDC1, NDUFS5, FOXRED1, ACAD9, NDUFA13, NDUFS2, LYRM2, NDUFAF2, OXA1L, NDUFS6, NDUFS8, NDUFAF3, NDUFC2, NUBPL
Complex I assembly	28	NDUFAF4, TMEM186, NDUFB6, NDUFS3, NDUFA8, NDUFC1, TIMMDC1, NDUFS5, NDUFV1, FOXRED1, NDUFB2, ACAD9, NDUFA13, NDUFB7, NDUFAB1, NDUFS2, NDUFAF2, NDUFA5, NDUFB10, NDUFB3, NDUFB4, NDUFB9, NDUFS6, NDUFAF3, NDUFC2, NUBPL, NDUFA1, NDUFV2
Complex II assembly	4	SDHA, SDHAF3, SDHAF1, SDHD
Complex III assembly	8	BCS1L, UQCC2, UQCC1, UQCRQ, UQCRC2, UQCRC1, CYC1, UQCRFS1

Gene set	n	Leading-edge genes
Complex IV assembly	19	NDUFA4, COX11, COX6C, COX20, COA3, COX7A2, HIGD1A, SMIM20, TACO1, CMC1, COX7C, COX5A, COX17, COX6A1, TMEM177, SURF1, COX6B1, COX5B, PET117
Mitochondrial translation	55	MRPL17, MRPL24, MRPL41, MRPL34, MRPS26, MRPS23, MTIF2, MRPL51, MRPS33, MRPL57, MRPS15, MRPL23, MRPL39, MRPL36, MRPS28, MRPL9, MRPL2, MRPS34, MRPS12, MRPL50, TSFM, GFM1, MRPL13, MRPL43, MRPL3, MRPS16, MRPS25, NDUFAB1, MRPL15, MRPS35, MRPS18B, OXA1L, MRPL20, TUFM, MRPL4, MRPL35, MRPL45, CHCHD1, MRPS17, MRPL37, ERAL1, MRPL38, MRPL22, MRPS7, MRPS21, MRPS22, MRPL40, MRPS14, MRPS9, MRPL12, MRPL46, MRPS18A, MRPS10, MTFMT, MRPL16

Table S2b-2. Leukocyte-poor RA vs OA.

Gene set	n	Leading-edge genes
ETC, NADH to ubiquinone	29	DNAJC15, NDUFC2, NDUFB6, NDUFA6, ISCU, NDUFAB1, NDUFA4, NDUFS2, NDUFA9, NDUFV2, NDUFA5, NDUFB4, NDUFB9, NDUFB2, NDUFB3, NDUFA7, NDUFS6, NDUFA11, NDUFB10, NDUFS3, NDUFS8, NDUFB8, NDUFS5, NDUFB7, NDUFC1, NDUFA1, NDUFA3, NDUFS7, DLD
NADH dehydrogenase assembly	17	TIMM21, NDUFC2, NDUFA6, BCS1L, NDUFS2, TMEM186, TIMMDC1, NDUFS6, NDUFS3, NDUFS8, FOXRED1, NDUFAF3, NDUFA13, NDUFAF4, NDUFS5, TMEM126B, NDUFS7
Complex I assembly	29	NDUFC2, NDUFA12, NDUFB6, NDUFA6, NDUFAB1, NDUFS2, TMEM186, NDUFV2, NDUFA5, NDUFB4, NDUFB9, NDUFB2, TIMMDC1, NDUFB3, NDUFA7, NDUFS6, NDUFB10, NDUFS3, FOXRED1, NDUFAF3, NDUFA13, NDUFAF4, NDUFB8, NDUFS5, NDUFB7, NDUFC1, TMEM126B, NDUFA1, NDUFA3
Complex II assembly	4	SDHAF4, SDHAF1, SDHA, SDHB
Complex III assembly	7	BCS1L, UQCRC1, UQCRQ, UQCC1, UQCRH, CYC1, UQCR11
Complex IV assembly	18	PET100, COX6C, COX6B1, COX7B, COX6A1, NDUFA4, CMC1, PNKD, COA3, COX5B, HIGD1A, COX8A, COX4I1, COX5A, COX7A2, COX11, COX17, COX14
Mitochondrial translation	54	MRPL41, MRPL11, CHCHD1, ERAL1, MRPS33, MRPL49, MRPS12, NDUFAB1, MRPL51, MALSU1, MRPL24, MRPL9, MTRF1, GFM2, MRPL36, MRPL20, MRPS15, MRPS16, MRPL13, MRPL45, MRPS23, MRPL43, MRPL44, MRPS18B, MRPL32, MRPS26, MRPL28, MTIF2, MRPL17, MRPL52, MRPL57, MRPL50, MRPL34, MRPL2, GADD45GIP1, MRPL21, MRPL23, MRPL54, MRPS34, MRPS18A, MRPS24, MRPS5, MRPL39, MRPS2, MRPS35, MRPL22, MRPL35, MRPL55, MRPL38, OXA1L, TUFM, MRPL15, MTFMT, MRPS14

Supplementary Figure S3.

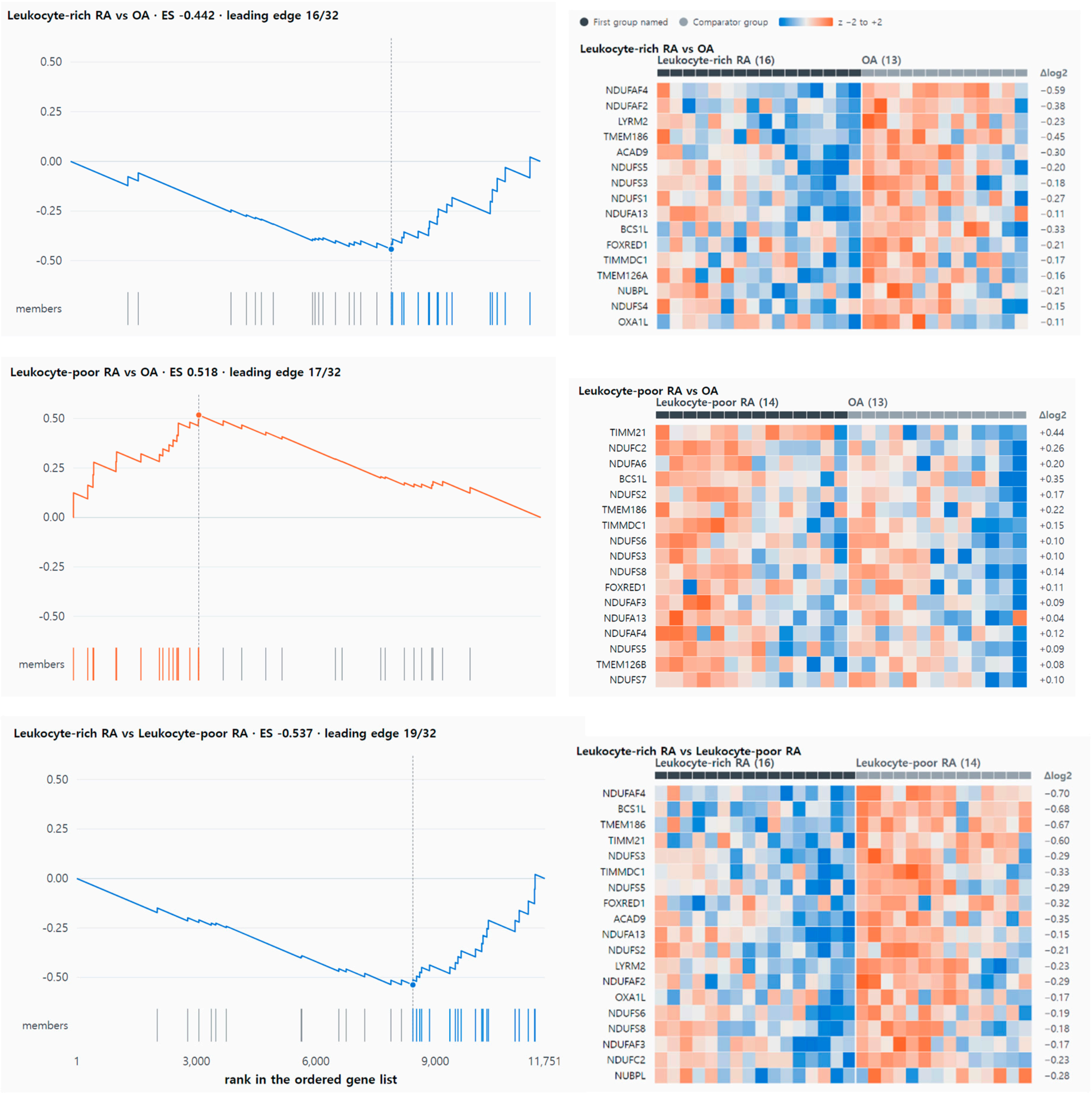

Supplementary Figure S3. NADH dehydrogenase complex assembly enrichment in synovial monocytes. Sorted monocytes, leukocyte-rich RA n = 16, leukocyte-poor RA n = 14, OA n = 13. Running enrichment score against genes ranked by Welch *t*. Leading-edge genes of the contrast at left, z-scored per gene; Δlog2, difference in mean log2TPM. Pre-ranked GSEA, 2,000 gene-set permutations.
